# Determination of the prebiotic activity of wheat arabinogalactan peptide (AGP) using batch culture fermentation

**DOI:** 10.1007/s00394-019-01908-7

**Published:** 2019-02-06

**Authors:** Suzanne Harris, Stephen Powers, Andrea Monteagudo-Mera, Ondrej Kosik, Alison Lovegrove, Peter Shewry, Dimitris Charalampopoulos

**Affiliations:** 1 Department of Food and Nutritional Sciences, University of Reading, Whiteknights, PO Box 226, Reading, RG6 6AP UK; 2 Department of Plant Science, Rothamsted Research, Harpenden, Hertfordshire AL5 2JQ UK; 3 Computational and Analytical Science, Rothamsted Research, Harpenden, Hertfordshire AL5 2JQ UK

**Keywords:** Arabinogalactan-peptide (AGP), Prebiotic, Batch culture, Fluorescence in situ hybridisation (FISH), Short chain fatty acids (SCFA)

## Abstract

**Purpose:**

To test the prebiotic activity of wheat arabinogalactan-peptide (AGP), which is a soluble dietary fibre composed of arabinogalactan polysaccharide linked to a 15-residue peptide, which accounts for up to 0.4% of the dry weight of wheat flour.

**Methods:**

The prebiotic activity of AGP prepared from white wheat flour was tested using in vitro fermentation by colonic bacteria in automated pH-controlled anaerobic stirred batch cultures and compared to fructooligosaccharide (FOS) and wheat flour arabinoxylan (AX). Bacterial populations were measured using fluorescence in situ hybridisation (flow-FISH) and short chain fatty acid (SCFA) concentrations were measured using HPLC.

**Results:**

Fermentation of AGP resulted in a significant bifidogenic activity and increased concentrations of SCFAs, mainly acetate after 24 h of fermentation.

**Conclusions:**

These results were comparable to those obtained with AX and confirm the prebiotic potential of AGP. Furthermore, fermentation of a mixture of AGP and AX was faster compared to the single substrates and more similar to FOS, indicating that combinations of fermentable carbohydrates with different structures are potentially more effective as prebiotics than single substrates.

**Electronic supplementary material:**

The online version of this article (10.1007/s00394-019-01908-7) contains supplementary material, which is available to authorized users.

## Introduction

Cereals are the most important source of dietary fibre (DF) in the human diet, providing about 40% of the total dietary intake in the UK, with bread contributing about half of this.

A number of definitions of dietary fibre have been proposed, the most widely used being that from the Codex Alimentarius 2009 which states that “dietary fibre consists of carbohydrate polymers with 10 or more monomeric units, which are not hydrolysed by the endogenous enzymes in the small intestine”. However, a footnote allows national authorities to also include “carbohydrates of 3–9 monomeric units” and these are usually included when considering wheat fibre. A number of studies have demonstrated that DF, and particularly cereal DF, has health benefits including regulation of satiety and diluting the energy density of food. The addition of insoluble DF to the diet increases stool weight from fibre bulk and increases in bacteria and water holding capacity. Soluble DF has also been shown to reduce the glycaemic index of food products, reduce insulin sensitivity and decrease cholesterol absorption. Furthermore, DF has also been shown to reduce the risk of colorectal cancer.

While whole wheat grain contains 11.5–15.5% total DF, the content is much lower in the white flour which is used to make most food products and comprises the starchy endosperm, but not the fibre-rich aleurone and outer layers of the grain. The major DF components in wheat are cell wall polysaccharides, which account for about 2–3% of the dry weight comprising about 70% arabinoxylan (AX), 20% (1 → 3,1 → 4)-β-d-glucan (β-glucan), 2% cellulose ((1 → 4)-β-d-glucan) and 7% glucomannan [1] and 1.4–1.7% fructo-oligosaccharides (fructans) [2]. In addition, white wheat flour contains up to 0.4% dry weight of arabinogalactan-peptide (AGP) [3, 4] which comprises a 15-residue amino acid peptide [5] including three hydroxyprolines which are *o*-glycosylated with branched arabinogalactan chains [6]. In most plants, arabinogalactans occur in covalent association with protein, either as proteoglycans or as glycoproteins, however, in wheat AGP, the polysaccharide is estimated to account for about 90% of the molecular mass. Although a recent study indicates that AGP is located in the cytoplasm or vacuole of the wheat cell, it does not appear to be essential for grain development and little is known of its biological function [7] or impact on human nutrition and health.

The process of fermentation, where colonic microbiota break down carbohydrates to monosaccharides before metabolising them to short chain fatty acids (SCFAs) appears to be particularly important to health benefits of DF. These benefits have led to the concept of “prebiotics”: substrates that are selectively utilized by host microorganisms conferring a health benefit [8]. Prebiotics can also alter the host colonic microbiota to a more favourable composition, for example, by increasing the proportions of beneficial bacteria (e.g., bifidobacteria and/or lactobacilli) [9].

Cereal DF components, particularly β-glucan and fructans, have well-established prebiotic activity, while a number of studies have demonstrated prebiotic activity for wheat AX [10–13]. However, although the concentration of AGP in wheat flour is similar to those of water-soluble AX and total β-glucan, its prebiotic potential has not been determined. We have, therefore, evaluated the prebiotic properties of AGP and determined whether AGP behaves synergistically with soluble AX from wheat flour, using an in vitro faecal culture system.

## Materials and methods

### Materials

AGP and water-soluble AX [average DP 131 (obtained using HP-SEC-MALLS using OHpak SB 802.5 HQ column on an Agilent 1260 infinity LC system)] were prepared from white flour from the wheat cultivar Yumai 34 using the method from Loosveld et al. [3] Fructo-oligosaccharides (FOS) from chicory (F8052 Sigma) (average DP 2–8) was used as a standard.

### Monosaccharide analysis

Fifty µL of a solution of 1 mg/mL AX was dried under vacuum to which was added 400 µL of 2M trifluoroacetic acid (TFA) and incubated at 120 °C for 1 h in a heating block to hydrolyse samples. Hydrolysed samples were cooled on ice and dried in speed-vac at 30 °C (overnight). 500 µL of water was added to remove any remaining TFA and the sample was dried again in the speed-vac. The sample was finally resuspended in 400 µL of MilliQ water. The hydrolysate was diluted further 1:1 with water. Standard curves were constructed for fucose, rhamnose, arabinose, galactose, glucose, xylose, mannose, galacturonic acid, and glucuronic acid using monosaccharide standards prepared from stock solutions of 1 mM and subjecting them to the same acid-hydrolysis protocol as for samples. All samples and standards were run under the same conditions as described below. Twenty µL was injected onto a Carbopac PA20 column with flow rate 0.5 mL/min and gradient: isocratic 4.5 mM KOH, 0–13 min; linear 4.5 to 10 mM KOH, 13–14 min; linear 10 to 13 mM KOH, 14–15 min; linear 13 to 20 mM, 15–16 min; isocratic 20 mM 16–17 min; linear 20 to 4.5 mM KOH, 17–18 min followed by isocratic 4.5 mM KOH 18–23 min; on a Dionex 5000 Ion Chromatography HPLC equipped with disposable gold electrode.

### MALDI–ToF-MS

MALDI–ToF-MS was as described in Marsh et al. [14] using a Micromass MALDI-LR mass spectrometer (Waters, Manchester, UK).

### In vitro fermentation

100-mL sterile batch fermentation vessels (50 mL working volume) were aseptically filled with 45 mL of sterile basal medium and sparged with O_2_- free N_2_ overnight to establish anaerobic conditions. The medium contained per litre: 2 g of peptone water (Oxoid Ltd., Basingstoke, UK), 2 g of yeast extract (Oxoid), 0.1 g of NaCl, 0.04 g of K_2_HPO_4_, 0.01 g of MgSO_4_·7H_2_O, 0.01 g of CaCl_2_·6H_2_O, 2 g of NaHCO_3_, 0.005 g of hemin (Sigma), 0.5 g of l-cysteine HCl (Sigma), 0.5 g of bile salts (Oxoid), 2 mL of Tween 80, 10 µL of vitamin K (Sigma). Polysaccharide samples were added (1% w/v) to the basal medium. Each vessel was inoculated with 10% (v/v) of faecal slurry from a single donor, which was prepared by homogenizing fresh human faeces (10%, w/w) in phosphate-buffered saline (PBS; 8 g/L NaCl, 0.2 g/L KCl, 1.15 g/L Na_2_HPO_4_, and 0.2 g/L KH_2_HPO_4_), pH 7.3 (Oxoid), using a stomacher (Stomacher 400, Seward). Three non-pooled faecal donors were used per experiment, two male and one female, between 23 and 59 years of age and on a normal diet without any special dietary requirements and that had not taken antibiotics, prebiotic or probiotics in the previous 3 months. Two experiments were run due to limitations of vessel numbers, one with a negative control (no carbon source added), FOS (0.5 g) (positive control), AGP (0.5 g) and AGP + AX (0.25 g and 0.25 g), and the second with a negative control, positive control as before and AX (0.5 g). Vessels were incubated at 37 °C with a water jacket for up to 48 h and the pH was controlled between 6.7 and 6.9 using an automated pH controller with 0.5 M HCL and NaOH (Fermac 260, Electrolab, Tewkesbury, UK). Samples 2 × 1 mL were collected at 0, 8 and 24 h for analysis.

### SCFA analysis using HPLC

Aliquots of 750 µL were removed from in vitro fermentation vessels and centrifuged at 13,000×*g* for 5 min to remove particulate matter and filtered using a 0.2 µM nitrocellulose filter. 20 µL was injected on to a Rezex ROA Organic Acid H^+^ (8%) HPLC column (Phenomenex, UK) at 50 °C on a Shimadzu Prominence HPLC with 0.0025 M H_2_SO_4_ eluent at a flow rate of 0.6 mL/min. SCFA (lactate, formate acetate, propionate and butyrate) were quantified with reference to calibration curves from 5 to 50 mM of authentic standards (Sigma).

### Enumeration of bacteria by flow-FISH

Samples of 750 µL removed from in vitro fermentation vessels were immediately placed on ice, before centrifugation at 13,000×*g* for 3 min and the supernatant discarded. Pelleted bacteria were fixed for 4 h at 4 °C in (PBS) and 4% (w/v) filtered paraformaldehyde (PFA) (Sigma-Aldrich P6148, pH 7.2) in a ratio of 1:3 (v/v). Samples were washed twice with filtered PBS and resuspended in 600 µL of a mixture of PBS/ethanol (1:1, v/v) and then stored at − 20 °C for up to 3 months. Hybridisation was carried out as described in Rycroft et al. [15, 16] using genus and group specific 16S rRNA-targeted oligonucleotide probes (MWG Biotech, Ebersberg, Germany).

The sample probes used were Bif164 [17], Bac303 [18], Lab158 [19], Ato291 [20], Prop853 [21], Erec482 [22], Rrec584 [21], Fprau655 [23], Chis150 [22], shown in Supplementary Table 1. Samples were screened using a flow cytometer (Accuri C6, BD Biosciences, USA) with Accuri CFlow software.

### Statistical analysis

The Genstat (2015, 18th edition, © VSN International Ltd, Hemel Hempstead, UK) statistical package was used for all analysis. One-way analysis of variance (ANOVA) and *F* test were applied to determine differences between treatments. Differences were deemed significant when *P* < 0.05.

## Results

### Monosaccharide analysis

Monosaccharide analysis of the AGP prepared from white flour (*Triticum aestivum* cv. Yumai 34) indicated that arabinose and galactose together comprised 96.73% (± 0.18) of total monosaccharides, with small amounts of glucose (2.6%) and xylose (1.74%). The A: G ratio for AGP was 0.48. The combined contents of arabinose and xylose in the arabinoxylan fraction prepared from the same flour were 91% (± 0.05), with galactose (5%) and glucose (4%). The A:X ratio for AX was 0.62. These data indicate that the AGP and AX fractions were over 95% and over 90% pure, respectively.

**Fig. 1:**
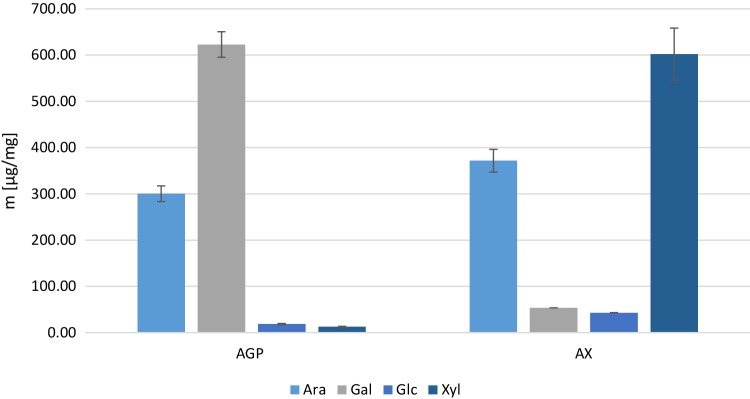
Monosaccharide analysis of extracted AGP and AX using a Carbopac PA20 column (*N* = 3). Error bars are SD

MALDI–TOF-MS was used to confirm the structure and purity of the carbohydrate moiety of the AGP, based on the molecular masses of the oligosaccharides released by the exo-b-(1 → 3)-galactanase. All samples were permethylated as described in Tryfona et al. [6] based on Ciucanu and Kerek [24] prior to mass spectrometry. Figure 1 shows the spectrum from 400 to 2400 *m*/*z*; the oligosaccharide composition is indicated by Hex (hexose residues) or Pent (pentose residues) while the subscript indicates the number of residues present, if greater than 1. The dominant ion was ‘Hex_2_ Pent’, at 637.5 *m*/*z* which is predicted to be two galactose units and an arabinose unit. The other ions are predicted as follows: *m*/*z* 477.7, Hex_2_; 841.5, Hex_3_Pent; 1001.6, Hex_3_Pent_2_; 1161.6, Hex_3_Pent_3_; 1365.8, Hex_4_Pent_3_; 1730.0, Hex_5_Pent_4_; 1934.0, Hex_6_Pent_4;_ 2095.2, Hex_6_Pent_5_.

### Effect of fermentation on SCFA concentrations

The concentrations of SCFA and lactate after fermentation of AGP were compared with the negative control (no substrate), FOS (positive control) and AX in Table 1. Significant increases (*p* < 0.05) compared to the negative control, occurred in the concentrations of total SCFAs for all substrates, which mainly resulted from increased acetic acid. Acetic acid concentrations increased after 8 h fermentation of all substrates, with FOS having the greatest increase. Acetate continued to increase until 24 h fermentation for all substrates, however, at 24 h the greatest increase in acetate was by fermentation of AGP (39.34 mM) and AGP + AX (38.91 mM). Large decreases occurred in lactate concentrations with the AGP, AGP + AX and AX substrates after 24 h compared with their negative controls. Total SCFA concentrations after 24 h fermentation were all significant and similar, the highest being with FOS (72.36 mM), then in order of decreasing concentration, AGP + AX (67.42 mM), AGP (61.95 mM) and AX (57.15 mM). Although the 1:1 mixture of AX and AGP resulted in higher concentrations of total SCFAs than either single substrate, these increases were not statistically different.

**Table 1:** SCFA and lactate concentration in batch cultures at 0, 4, 8 and 24 h fermentation comparing no substrate, FOS, AGP and AX

	Time	Lactate	Acetate	Propionate	Butyrate	Total
Negative control	0	2.28 (1.20)	3.13 (1.05)	3.52 (0.62)	2.86 (1.08)	21.61 (1.48)
	8	6.30 (1.79)	8.31 (1.84)	5.10 (0.97)	1.57 (0.65)	27.12 (5.41)
	24	10.17 (2.42)	8.28 (2.04)	10.25 (5.19)	3.32 (1.24)	36.33 (8.49)
FOS	0	7.22 (3.51)	9.16 (1.09)	5.27 (1.80)	2.33 (0.28)	27.55 (8.28)
	8	12.61 (8.95)	30.57 (4.77)*	11.27 (5.59)	3.09 (1.98)	67.45 (17.12)*
	24	10.16 (5.53)	33.35 (6.69)*	13.03 (7.03)	8.36 (6.59)	72.36 (32.92)*
AGP	0	7.86 (5.12)	6.80 (3.25)	3.49 (1.52)	5.78 (1.67)	25.54 (5.97)
	8	5.75 (2.37)	23.83 (5.95)*	12.33 (5.09)	2.81 (1.07)	50.96 (11.40)*
	24	3.48 (1.65)*	39.34 (4.53)*	8.60 (6.90)	5.20 (3.53)	61.95 (9.94)*
AGP + AX	0	10.01 (2.58)	6.95 (3.49)	5.48 (1.37)	3.53 (0.84)	36.92 (9.28)
	8	4.50 (1.98)	17.05 (8.52)	12.86 (5.25)	2.37 (1.94)	44.08 (15.81)
	24	2.38 (1.03)*	38.91 (9.58)*	15.61 (6.47)	2.95 (1.74)	67.42 (10.47)*
Negative control for AX	0	11.02 (3.68)	4.21 (1.22)	4.36 (1.11)	2.22 (0.50)	21.85 (3.15)
	8	5.66 (0.44)	9.05 (2.40)	8.77 (1.42)	1.53 (0.36)	26.24 (3.97)
	24	6.45 (0.16)	9.84 (2.71)	9.33 (1.85)	1.20 (0.22)	30.08 (1.97)
AX	0	16.39 (3.37)	4.93 (1.01)	5.05 (0.60)	1.54 (0.20)	27.91 (3.04)
	8	10.19 (2.86)	21.12 (4.46)*	8.09 (0.48)	1.76 (1.76)	47.79 (4.99)*
	24	2.56 (1.31)*	27.64 (4.85)*	9.76 (2.77)	1.29 (0.12)	57.15 (12.25)*

Formate is included in total SCFA but not shown. One-way AVONA was applied to the data to test the main interaction between treatments. Significant interaction between treatments and negative control are denoted. Standard error of the mean (SEM) is shown in brackets. Significant differences between treatments and relevant negative control are denoted *p = 0.05 (*F* test)

### Effect of fermentation on bacterial populations

**Fig. 2:**
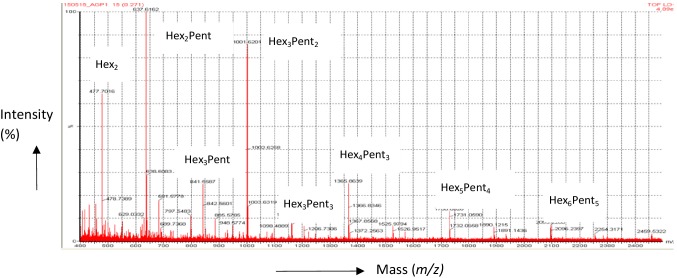
MALDI–ToF-MS spectra showing ions of *m*/*z* diagnostic of per-methylated oligosaccharides released from AGP by exo-B-(1 → 3) galactanase digestion. AGP was isolated from *Triticum aestivum* cv. Yumai-34 white flour. Spectra shows 400–2400 *m*/*z*

The populations of the dominant types of human colonic bacteria are shown in Table 2, while the populations of total enumerated bacteria, *Bifidobacterium* and *Clostridium coccoides*/*Eubacterium rectale* are shown in Fig. 2. The *Bifidobacterium* populations increased significantly (*p* < 0.05) compared to the negative control after 8 h fermentation for FOS (positive control) and AGP + AX with the greatest increase of 1.95 log occurring with fermentation of FOS, followed by an increase of 1.37 log with AGP + AX. The populations of *Bifidobacterium* increased with the separate AX and AGP substrates between 8 and 24 h, but decreased 0.88 log between 8 and 24 h for FOS and 0.53 log for AGP + AX. As with *Bifidobacterium*, the *Clostridium coccoides–Eubacterium rectale* group increased after 8 h fermentation of FOS and AGP + AX, and after 24 h fermentation of AX and AGP. No significant changes were observed in the *Lactobacillus Enterococcus* group, *Bacteroides–Prevotella* group, *Roseburia, Atopobium, Desulfovibrionales, Clostridium* cluster IX, *Faecalibacterium prausnitzii* group or *Clostridium*-cluster I and II. The 1:1 mixture of AGP and AX gave significantly greater populations of the beneficial *Bifidobacterium* and *Clostridium coccoides*/*Eubacterium rectale* groups than either single substrate at 8 h, but these were lower at 24 h.

**Table 2:** Bacterial enumeration of in vitro batch culture fluid after fermentation comparing no substrate, FOS, AGP and AX

	Time (h)	*Bifidobacterium* genus	*Lactobaccillus Enterococcus* group	*Bacteroides- Prevotella* group	*Clostridium coccoides- Eubacterium rectale* group	*Roseburia*	*Atopobium* cluster	*Clostridium* cluster IX	Faecalibacterium prausnitzii group	*Desulfovibrionales*	*Clostridium-cluster I and II*	Total
Negative	0	7.96 (0.26)	6.80 (0.38)	7.14 (0.031)	8.53 (0.33)	6.78 (0.75)	7.20 (0.58)	7.35 (0.36)	8.28 (0.25)	7.85 (0.47)	6.86 (0.40)	8.97 (0.33)
	8	7.72 (0.11)	7.16 (0.12)	7.43 (0.22)	8.37 (0.25)	7.38 (0.02)	7.20 (0.10)	7.42 (0.27)	8.06 (0.24)	7.69 (0.30)	7.31 (0.17)	8.85 (0.22)
	24	7.68 (0.66)	7.25 (0.73)	7.36 (0.68)	8.00 (0.49)	7.46 (0.71)	7.51 (0.52)	7.53 (0.60)	7.49 (0.58)	7.38 (0.46)	7.12 (0.73)	8.62 (0.57)
FOS	0	7.88 (0.27)	7.01 (0.46)	7.57 (0.43)	8.23 (0.27)	7.41 (0.50)	7.01 (0.49)	7.45 (0.35)	8.22 (0.25)	7.75 (0.46)	7.11 (0.59)	8.87 (0.30)
	8	9.83 (0.15)*	7.32 (0.60)	8.20 (0.40)	9.70 (0.17)*	8.16 (0.70)	8.02 (0.46)	8.22 (0.38)	8.31 (0.41)	7.88 (0.55)	7.69 (0.48)	10.26 (0.09)*
	24	8.95 (0.21)	7.13 (0.72)	7.43 (0.66)	8.73 (0.20)	7.29 (0.72)	7.17 (0.54)	7.72 (0.60)	7.89 (0.58)	7.61 (0.60)	7.16 (0.60)	9.36 (0.27)
AGP	0	7.96 (0.25)	7.02 (0.26)	7.23 (0.10)	8.59 (0.19)	7.66 (0.24)	7.27 (0.30)	7.41 (0.17)	8.19 (0.24)	8.04 (0.19)	7.16 (0.19)	9.00 (0.20)
	8	8.89 (0.45)	6.87 (0.51)	6.74 (0.70)	8.92 (0.36)	7.89 (0.22)	7.23 (0.14)	7.32 (0.30)	8.47 (0.16)	8.06 (0.08)	7.18 (0.35)	9.49 (0.31)
	24	9.39 (0.78)*	7.26 (0.33)	7.68 (0.83)	9.28 (0.47)*	7.46 (0.30)	7.26 (0.37)	7.89 (0.78)	8.03 (0.17)	7.19 (0.35)	6.24 (0.76)	9.84 (0.55)*
AGP + AX	0	7.98 (0.18)	7.06 (0.36)	7.20 (0.46)	8.63 (0.29)	7.37 (0.46)	7.12 (0.46)	7.20 (0.41)	8.38 (0.19)	7.95 (0.36)	7.04 (0.40)	9.06 (0.26)
	8	9.35 (0.46)*	7.22 (0.44)	7.74 (0.28)	9.36 (0.38)*	7.61 (0.78)	7.00 (0.12)	7.63 (0.31)	8.59 (0.23)	8.00 (0.35)	6.94 (0.30)	9.89 (0.33)*
	24	8.82 (0.28)	6.88 (0.76)	6.73 (0.67)	8.82 (0.19)	6.64 (0.75)	6.27 (0.28)	6.47 (0.53)	7.13 (0.30)	6.07 (0.45)	5.62 (0.43)	9.25 (0.13)
Negative for AX	0	8.53 (0.33)	8.76 (0.13)	7.95 (0.12)	9.11 (0.04)	8.63 (0.11)	7.60 (0.16)	8.20 (0.09)	9.07 (0.04)	8.61 (0.06)	7.75 (0.15)	9.70 (0.03)
	8	8.58 (0.32)	7.65 (0.03)	8.55 (0.03)	9.01 (0.08)	8.25 (0.13)	7.70 (0.09)	8.70 (0.06)	8.90 (0.02)	8.54 (0.10)	8.20 (0.12)	9.67 (0.07)
	24	8.76 (0.3)	7.90 (0.18)	8.45 (0.13)	8.92 (0.07)	7.74 (0.20)	7.85 (0.10)	8.76 (0.08)	8.54 (0.06)	8.12 (0.12)	7.93 (0.12)	9.58 (0.10)
AX	0	8.52 (0.41)	7.92 (0.14)	8.11 (0.17)	9.13 (0.01)	8.65 (0.06)	7.88 (0.05)	8.28 (0.12)	8.89 (0.09)	8.58 (0.03)	8.00 (0.16)	9.67 (0.07)
	8	9.23 (0.19)*	7.67 (0.20)	8.84 (0.23)	9.09 (0.38)	8.32 (0.52)	7.65 (0.37)	8.93 (0.24)	9.00 (0.19)	8.61 (0.19)	7.81 (0.40)	9.94 (0.24)*
	24	9.84 (0.09)*	8.08 (0.22)	8.55 (0.44)	9.57 (0.18)*	8.42 (0.40)	8.45 (0.53)	8.63 (0.44)	9.06 (0.17)	8.26 (0.20)	8.11 (0.42)	10.29 (0.05)*

Negative control is no added carbohydrate and positive control is FOS. Values are mean log_10_ bacterial numbers/mL found using flow FISH. One-way AVONA was applied to the data to test the main interaction between treatments. Values in brackets are SEM. Significant difference between treatments and relevant negative control are denoted *p = 0.05 (*F* test)

## Discussion

This study aimed to determine the prebiotic potential of the soluble wheat fibre AGP. AGP isolated from wheat flour was characterised and evaluated for prebiotic activity based on increases in the populations of beneficial bacteria and in the production of SCFA, using in vitro batch cultures. The fermentation of AGP was also compared to FOS and AX, which have established prebiotic activity [10, 11, 25, 26], in addition, a mixture of AGP and AX was tested to determine whether the combination may result in a synergistic prebiotic effect.

Short chain fatty acids (SCFA) are volatile fatty acids consisting of a straight-chain, aliphatic tail of fewer than six carbon atoms and are produced by fermentation of oligosaccharide concomitant with increases in beneficial bacteria including *Bifidobacterium*. The principal SCFAs are acetate, propionate and butyrate, together comprising 95% of all SCFAs produced [27] and are metabolized primarily by the colonic epithelium (butyrate), liver (propionate) and muscle (acetate) [28]. The concentrations of SCFAs in this study were used as a measure of the rate of fermentation of the substrates, with significant increases particularly apparent in the predominant SCFA, acetate. The spectra in Fig. 2 are very similar to those reported for AGP from white flour of cv. Cadenza by Tryfona et al. [6]. The mass spectra, therefore, confirm the purity and identity of the AGP used for in vitro fermentation.

**Fig. 3:**
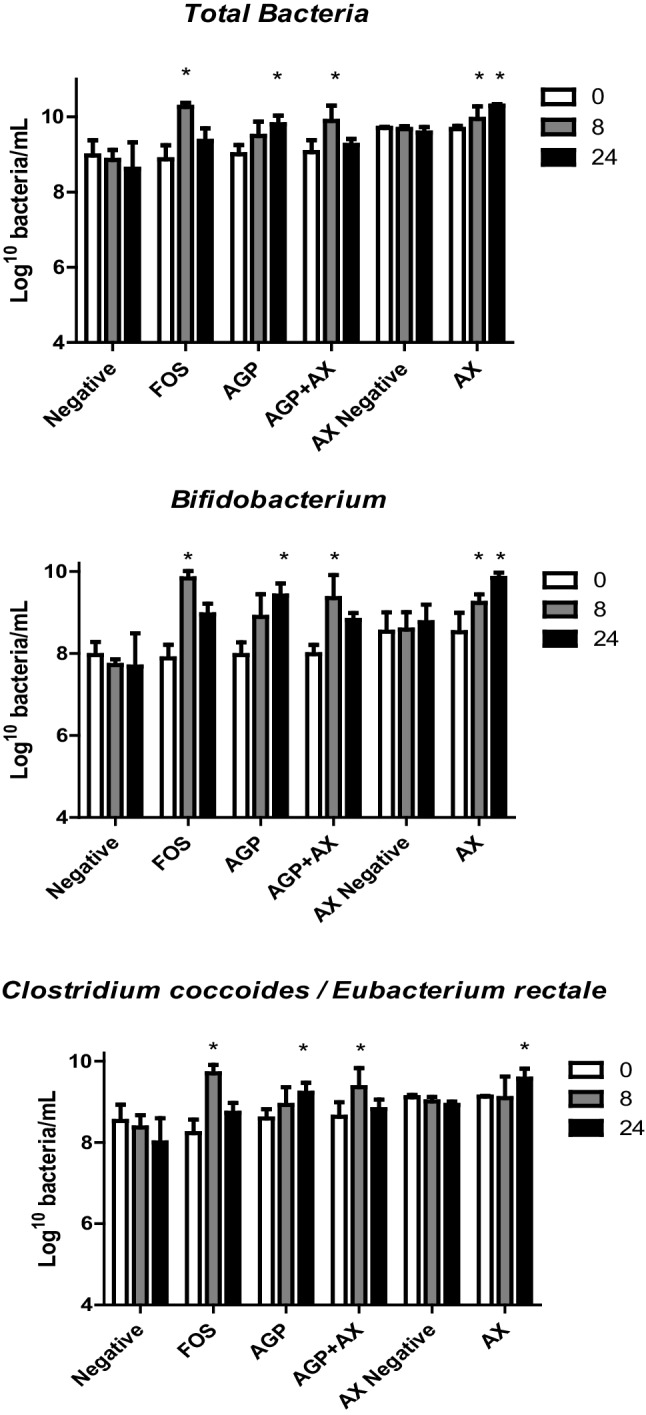
Total bacteria, *Bifidobacterium* and *Clostridium coccoides*/ *Eubacterium rectale* populations after fermentation of different substrates at times 0, 8, and 24 h analysed by Flow-FISH. Error bars show SEM (*n* = 3). Significant differences (*p* < 0.05) from negative control are denoted with asterisk

Despite the huge variety of different bacterial populations present in the gut and relatively low numbers of the bacterial genus *Bifidobacterium* in the healthy adult (< 5%) [29] this genus is most often targeted by prebiotics. This is because of it’s association with multiple health benefits, including reducing the proliferation of colorectal cancer and the concentration of circulating cholesterol [30, 31]. A decrease *Bifidobacterium* levels below those in healthy adults has been linked to disorders such as antibiotic-associated diarrhoea, inflammatory bowel disease, irritable bowel syndrome, obesity and allergies [32] demonstrating their importance in the colon despite relatively low numbers. In this study, all substrates demonstrated beneficial effects by significantly increasing (*p* < 0.05) the populations of *Bifidobacterium* from 8 to 24 h compared to the negative control (Table 2; Fig. 3). Unlike the FOS and AGP + AX mixture which showed the maximum *Bifidobacterium* growth at 8 h, proliferation was slower with AGP and AX singly as substrates, reaching the greatest population numbers after 24 h. This effect was observed with all donors in the study; therefore, it appears to show that bifidobacteria ferment soluble wheat flour AX and AGP more slowly than FOS. The same effect was observed with the populations of the predominant beneficial bacterial group [33] *Clostridium coccoides*/*Eubacterium rectale* (*Clostridium* Cluster XIVa and XIVb), which showed significant increases simultaneously with bifidogenic effects and may be indicative of cross feeding interactions as reported by Rivière et al. [32].

The structures of fermentable carbohydrates, including the degree of polymerisation (DP) and molecular weight have previously been shown to affect the rate of fermentation [26] and FOS is thought to be rapidly fermented due to its low DP [34]. In this study, the DP of the AX (average DP 131) was much greater than that of FOS (DP 2–8). The longer polysaccharides in AX have fewer non-reducing ends per unit mass than FOS, providing less substrate for hydrolysis by bacterial enzymes, which may have contributed to the slower rate of fermentation shown with AX. Wheat AGP is considered have three carbohydrate moieties. Their molecular masses have not been determined but estimates of between 122 and 389 sugar residues can be made based on the reported mass of the whole AGP molecule, ranging from 22,000 to 70,000 [4, 35–37]. This mass is much greater than that of FOS, accounting for the slower fermentation.

A slower rate can be advantageous for health as it allows the prebiotic to reach the more distal regions of the colon, where the levels of fermentable carbohydrate are lower, and fermentation of proteins occurs with adverse effects [38].

The combination of AX + AGP showed faster fermentation than either substrate singly, with significant increases in beneficial bacterial populations by 8 h fermentation, similar to that of FOS.

It is possible that a faster fermentation may be achieved via utilization of multiple non-competing bacterial enzymes. For example, some *Bacteroides* spp. have been found to fully ferment highly branched xylans as well as β1–3 and β1–4 arabinogalactans from soy by producing multiple enzymes [39].

*Desulfovibrionales* (DSV) is a group of sulphate-reducing bacteria which are suggested to contribute to the development of ulcerative colitis through the production of cytotoxic H_2_S and add to the pathology of the disease [40, 41] (although this role is disputed as analyses of bacterial populations from faeces and mucosal biopsies have so far failed to demonstrate changes in DSV populations associated with the disease) [42]. Similarly, bacteria of *Clostridium cluster I* and *II* are also considered to have adverse effects on health, as they are associated with protein fermentation and some end products of protein fermentation can be harmful to the host, e.g., amines and ammonia [43]. A shift to protein fermentation has been linked with increases in diseases such as irritable bowel syndrome (IBS) and colonic cancers, which occur more often in the distal regions of the gut [43, 44]. The populations of *Desulfovibrionales*, and *Clostridium-cluster I and II* did not increase with any of the substrates, despite the presence of a peptide chain in the AGP. This could be due to competition from saccharolytic bacteria which were still increasing up to 24 h of fermentation, to the low proportion of the peptide in the AGP structure 8% [45] or to the inaccessibility of the peptide, surrounded by arabinogalactan [45].

Total SCFA concentrations were highest with the positive control (FOS) after 24 h and comprised mostly acetate. The second highest concentration of total SCFAs was generated by AGP + AX combined, being higher than those resulting from fermentation of either single component and comprised mainly acetate and propionate. The most abundant SCFAs, acetate, propionate and butyrate, have been shown to have multiple beneficial effects for the host, for example, by providing dietary energy, and by suppressing the growth of pathogens by decreasing the pH of the intestinal lumen [46]. These SCFAs were also reported to have anti-inflammatory effects in rats [47] and influence intestinal motility in rats via G-protein coupled receptor activation, with acetate being the most effective, followed by propionate and butyrate [48]. The production pathways of acetate are found widely among bacterial groups, however, pathways for production of propionate, butyrate, and lactate appear more highly conserved and substrate specific [49].

Large increases in acetate were observed after fermentation of all substrates, with AGP and AGP + AX showing the greatest increases. Bifidobacteria are known to produce acetate [50, 51] and were observed to increase concomitantly with acetate concentration with all substrates, however, (as Actinobacteria) they are present in much smaller numbers than bacteria from the Bacteroides and Firmicutes phyla. Acetate production occurs via widely distributed pathways among bacterial groups so the increases in acetate can also be attributed to other bacteria, including the predominant group found in the gut which can also produce acetate, the *Clostridium coccoides* group [33, 52] which increased in all substrates. Pathways for propionate, butyrate and lactate production appear more highly conserved and substrate specific [49]. The decreases in lactate observed during fermentation of AGP, AGP + AX and AX demonstrate a healthy colonic environment and bacterial cross feeding. Under healthy gut conditions lactate is only present in low concentrations in faeces (< 5 mM) [53] because bacterial breakdown markedly exceeds production [54]. Lactate is formed from pyruvate through the action of lactate dehydrogenase in the homofermentative pathway by many common gut bacteria including *Lactobacillus, Bifidobacterium, Enterococcus*, and *Streptococcus* and *Eubacterium* spp. [55], but can also be converted to other SCFA. Decreases in lactate can, therefore, represent cross-feeding of different bacterial species including the species *Roseburia intestinalis, Eubacterium rectale, Eubacterium halii*, and *Anaerostipes caccae* [53, 54, 56, 57] which utilise lactate for production of other SCFAs-mainly butyrate, but also propionate and valerate [56]. Because this mechanism is widely utilised it is not possible to attribute the decreases in lactate to specific bacterial groups in this study, however, the large decreases in lactate shown by fermentation of both AGP and AGP + AX demonstrates a greater proportion of lactate-utilizing than lactate-producing bacteria which is important as an accumulation of lactate in the gut can cause acidosis, neurotoxicity, and cardiac arrhythmia [58]. Lactate levels were not observed to drop over time with fermentation of FOS, which remained similar to the negative control, however, this was due to large individual variations (Table S1).

Butyrate is produced by a range of bacteria, including the *Clostridium, Roseburia* and *Eubacterium* genera [51] but is dominated by *Faecalibacterium prausnitzii, Eubacterium rectale, Eubacterium hallii* and *R. bromii* [56]. No significant increases in butyrate were observed with fermentation of any of the substrates in this study (although FOS gave a non-significant increase by 24 h). It is thought that wheat polysaccharides, which would include, AX and AGP, are not directly butyrogenic, but rely on cross-feeding interactions between bacteria that utilize metabolites to produce butyrate and those producing the precursor metabolites directly from fermentation (e.g., *Eubacterium* spp., *Faecalibacterium prausnitzii*, and *Roseburia* which can utilize acetate from bifidobacteria) [58]. The butyrate concentration has previously been shown to increase during in vitro fermentation of several commercially available samples of wheat AX [23], however, this effect was not observed in this study and may be due to a lack of the dominant butyrate producers *Faecalibacterium prausnitzii* [56], which did not increase during fermentation.

Wheat AGP showed potential prebiotic activity during in vitro fermentation, by selectively increasing populations of beneficial bacteria including *Bifidobacterium* and *Eubacterium* genera and providing increases in the concentration of SCFAs (mainly consisting of acetate). A slower fermentation can demonstrate that a substrate is able to persist to more distal regions of the colon. AGP showed slower bacterial fermentation than FOS, however, this persistence is unlikely to occur when wheat products are consumed as combining AGP with AX resulted in faster utilisation of the substrates. Since the ratio of water-soluble AX to AGP used in these experiments is similar to that in white wheat flour, their potential to act synergistically is more relevant to the consumption of wheat products than the results obtained with single substrates. This study used faecal samples to provide microbial populations for fermentation in vitro. The results should, therefore, be confirmed with larger numbers of samples and an in vivo human intervention study to further clarify the role of AGP/AGP + AX in colonic fermentation.

## Electronic supplementary material

Below is the link to the electronic supplementary material.

Supplementary material 1 (DOCX 23 KB)
