# Supplementary material for: Determination of the prebiotic activity of wheat arabinogalactan peptide (AGP) using batch culture fermentation
Source: Eur J Nutr. 2019 Feb 6;59(1):297–307. doi: 10.1007/s00394-019-01908-7 (PMC7000537; doi:10.1007/s00394-019-01908-7)
Supplement: Supplementary file 1 — Supplementary material 1 (DOCX 23 KB) [file 394_2019_1908_MOESM1_ESM.docx]

Table S1 16S rRNA-targeted oligonucleotide probes

| **Probe Name** | **Sequence (5’ to 3’)** | **Fluorescence** | **Targeted Bacteria** | **Reference** |
| --- | --- | --- | --- | --- |
| Non Eub | ACTCCTACGGGAGGCAGC | Alexa-488 | None | Wallner et al., 1993 [62] |
| Eub338I* | GCTGCCTCCCGTAGGAGT | Alexa-488 | All | Daims et al., 1999 [63] |
| Eub338II* | GCAGCCACCCGTAGGTGT | Alexa-488 | All |  |
| Eub338III* | GCTGCCACCCGTAGGTGT | Alexa-488 | All |  |
| Non Eub | ACTCCTACGGGAGGCAGC | Alexa-647 | None | Wallner et al., 1993 [62] |
| Eub338I | GCTGCCTCCCGTAGGAGT | Alexa-647 | All | Daims et al., 1999 [63] |
| Eub338II | GCAGCCACCCGTAGGTGT | Alexa-647 | All |  |
| Eub338III | GCTGCCACCCGTAGGTGT | Alexa-647 | All |  |
| Bif164 | CATCCGGCATTACCACCC | Alexa-647 | *Bifidobacterium* | Langendijk et al., 1995 [28] |
| Lab158 | GGTATTAGCAYCTGTTTCCA | Alexa-647 | *Lactobacillus* and *Enterococcus* | Harmsen et al., 1999 [30] |
| Bac303 | CCAATGTGGGGGACCTT | Alexa-647 | *Bacteroides* | Manz et al., 1996 [29] |
| Erec482 | GCTTCTTAGTCARGTACCG | Alexa-647 | Eubacterium rectale/Clostridium coccoides cluster | Franks et al., 1998 [33] |
| Rrec584 | TCAGACTTGCCGYACCGC | Alexa-647 | *Roseburia* | Walker et al., 2005 [32] |
| Ato291 | GGTCGGTCTCTCAACCC | Alexa-647 | *Atopobium* | Harmsen et al., 2000 [31] |
| Prop853 | ATTGCGTTAACTCCGGCAC | Alexa-647 | *Clostridium cluster IX* | Walker et al., 2005 [32] |
| Fprau655 | CGCCTACCTCTGCACTAC | Alexa-647 | Faecalibacterium prausnitzii | Devereux et al., 1992 [64] |
| DSV687 | TACGGATTTCACTCCT | Alexa-647 | *Desulfovibrionales* | Hold et al., 2003 [34] |
| Chis150 | TTATGCGGTATTAATCTYCCTTT | Alexa-647 | *Clostridium histolyticum* | Franks et al., 1998 [33] |
